# Characterization and Expression of Genes Encoding Three Small Heat Shock Proteins in *Sesamia inferens* (Lepidoptera: Noctuidae)

**DOI:** 10.3390/ijms151223196

**Published:** 2014-12-12

**Authors:** Meng Sun, Ming-Xing Lu, Xiao-Tian Tang, Yu-Zhou Du

**Affiliations:** 1 School of Horticulture and Plant Protection & Institute of Applied Entomology, Yangzhou University, Yangzhou 225009, China; E-Mails: sunmeng8888@gmail.com (M.S.); lumx@yzu.edu.cn (M.-X.L.); tangxt1@hotmail.com (X.-T.T.); 2 Institute of Plant Protection, Shandong Academy of Agricultural Sciences, Jinan 250100, China

**Keywords:** *Sesamia inferens*, small heat shock protein, cloning, expression pattern

## Abstract

The pink stem borer, *Sesamia inferens* (Walker), is a major pest of rice and is endemic in China and other parts of Asia. Small heat shock proteins (sHSPs) encompass a diverse, widespread class of stress proteins that have not been characterized in *S. inferens*. In the present study, we isolated and characterized three *S. inferens* genes that encode members of the α-crystallin/sHSP family, namely, *Sihsp21.4*, *Sihsp20.6*, and *Sihsp19.6*. The three cDNAs encoded proteins of 187, 183 and 174 amino acids with calculated molecular weights of 21.4, 20.6 and 19.6 kDa, respectively. The deduced amino acid sequences of the three genes showed strong similarity to sHSPs identified in other lepidopteran insects. *Sihsp21.4* contained an intron, but *Sihsp20.6* and *Sihsp19.6* lacked introns. Real-time quantitative PCR analyses revealed that *Sihsp21.4* was most strongly expressed in *S. inferens* heads; Whereas expression of *Sihsp20.6* and *Sihsp19.6* was highest in eggs. The three *S. inferens* sHSP genes were up-regulated during low temperature stress. In summary, our results show that *S. inferens* sHSP genes have distinct regulatory roles in the physiology of *S. inferens*.

## 1. Introduction

Small heat shock proteins (sHSPs) were first identified as a set of low molecular proteins (15–30 kDa) induced after heat shock in *Drosophila melanogaster* [1]. Compared to other HSPs, sHSPs exhibit a greater variation in sequence, structure, size, and function [2,3]. They are a superfamily of proteins that contain an α-crystallin domain and variable *N*- and *C*-terminal extensions [4]. sHSPs can exist as large oligomers comprised of ~50 subunits and can approach a mass of 1.2 MDa [5,6]. Structurally, sHSPs remain poorly understood, mainly because members of this protein family are extremely dynamic and heterogeneous [7,8]. Many function as molecular chaperones that block the aggregation of unfolded proteins and have cytoprotective functions under stressful conditions [9]. In addition to the stress response, sHSPs have been implicated in apoptosis and autophagy, actin and intermediate filament dynamics, organization of the cytoskeleton, and membrane fluidity [10,11,12,13]. They also function as therapeutic targets and biomarkers for many diseases [14]. In insects, they presumably perform important roles in heat/cold stress, metamorphosis, normal development, diapause, and immune responses [15,16,17,18,19,20,21]; However, these roles remain for many insect species.

The pink stem borer, *Sesamia inferens* (Walker) (Lepidoptera: Noctuidae), is a major pest of rice in China and other parts of Asia, and recently, damage incited by *S. inferens* has become more serious [22]. According to our previous surveys, this pest now occurs in the more northern regions of China. Many studies of *S. inferens* have focused on biological characteristics [23,24,25,26,27,28]. We previously demonstrated that *S. inferens* still survived during exposure to low temperatures [29].

The underlying mechanisms that explain sudden outbreaks and the widespread distribution of *S. inferens* remain obscure. Hence, expression analysis of relevant genes, such as those encoding sHSPs, may provide insight on the incidence of *S. inferens*. To investigate whether *shsps* expression regulates cold tolerance in *S. inferens*, we cloned three genes encoding sHSPs from this insect pest. The structure of these genes was examined, and we analyzed their expression in different tissues and stages of insect development. Our results indicate that expression of the three *shsps* is modulated in response to cold stress.

## 2. Results and Discussion

### 2.1. Results

#### 2.1.1. Sequence Analysis of *S. inferens* Small Heat Shock Proteins (sHSPs) Genes

Three complete cDNA sequences were obtained and named *Sihsp21.4*, *Sihsp20.6*, and *Sihsp19.6*; These were deposited as GenBank accession nos. KM217075, KM217077, and KM217079, respectively. The sizes of full-length cDNAs were 1385 (*Sihsp21.4*), 835 (*Sihsp20.6*), and 798 bp (*Sihsp19.6*). Each gene contained three regions, a 5' untranslated region (5' UTR), the open reading frame (ORF), and a 3' UTR. Lengths of 5' UTRs were 118 (*Sihsp21.4*), 119 (*Sihsp20.6*), and 146 bp (*Sihsp19.6*), respectively. Lengths of 3' UTRs were 703 (*Sihsp21.4*), 164 (*Sihsp20.6*), and 127 bp (*Sihsp19.6*); Each 3' UTR contained a polyadenylation signal (AATAAA) located 16 (*Sihsp21.4*), 21 (*Sihsp20.6*), and 22 bp (*Sihsp19.6*) upstream with respect to the poly(A) tract.

**Figure 1:**
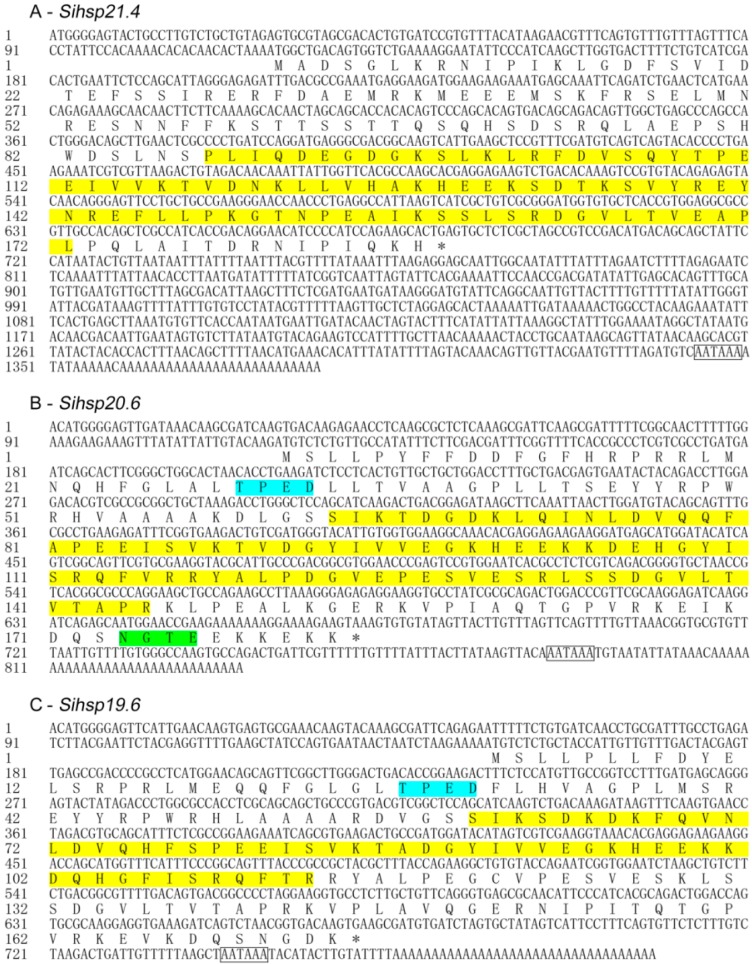
Nucleotide and deduced amino acid sequences of small heat shock proteins (sHSPs) genes from *Sesamia inferens*. sHSP genes include *Sihsp21.4* (**A**); *Sihsp20.6* (**B**); and *Sihsp19.6* (**C**). HSP20 family profiles, typical α-crystalline domains, are shaded in yellow; Casein kinase II phosphorylation sites (TPED) are shaded in cyan; *N*-Glycosylation site (NGTE) is shaded in green; The putative polyadenylation signals are boxed.

The three ORFs consisted of 564 (*Sihsp21.4*), 552 (*Sihsp20.6*), and 525 nucleotides (*Sihsp19.6*). The deduced proteins contained 187 (HSP21.4), 183 (HSP20.6), and 174 amino acids (HSP19.6), with predicted molecular weights of 21.4, 20.6, and 19.6 kDa, respectively. The theoretical isoelectric points (pI) of the predicted proteins were 5.79 (HSP21.4), 6.54 (HSP20.6), and 6.53 (HSP19.6). When compared with the NCBI GenBank and PROSITE databases, the three deduced proteins had high similarity to the small heat shock protein (HSP20) family. Each of the predicted proteins contained the typical α-crystallin domain at the following locations: HSP21.4, 88–172 aa (Figure 1A), HSP20.6, 63–145 aa (Figure 1B), and HSP19.6, 60–142 aa (Figure 1C). PROSITE analysis indicated that HSP20.6 has a casein kinase II phosphorylation site (TPED, 29–32 aa) and a *N*-glycosylation site (NGTE, 174–177 aa) (Figure 1B). Similarly, *S. inferens* HSP19.6 also contains a casein kinase II phosphorylation site (TPED, 27–30 aa) (Figure 1C).

#### 2.1.2. Structural Prediction of *S. inferens* sHSPs

**Figure 2:**
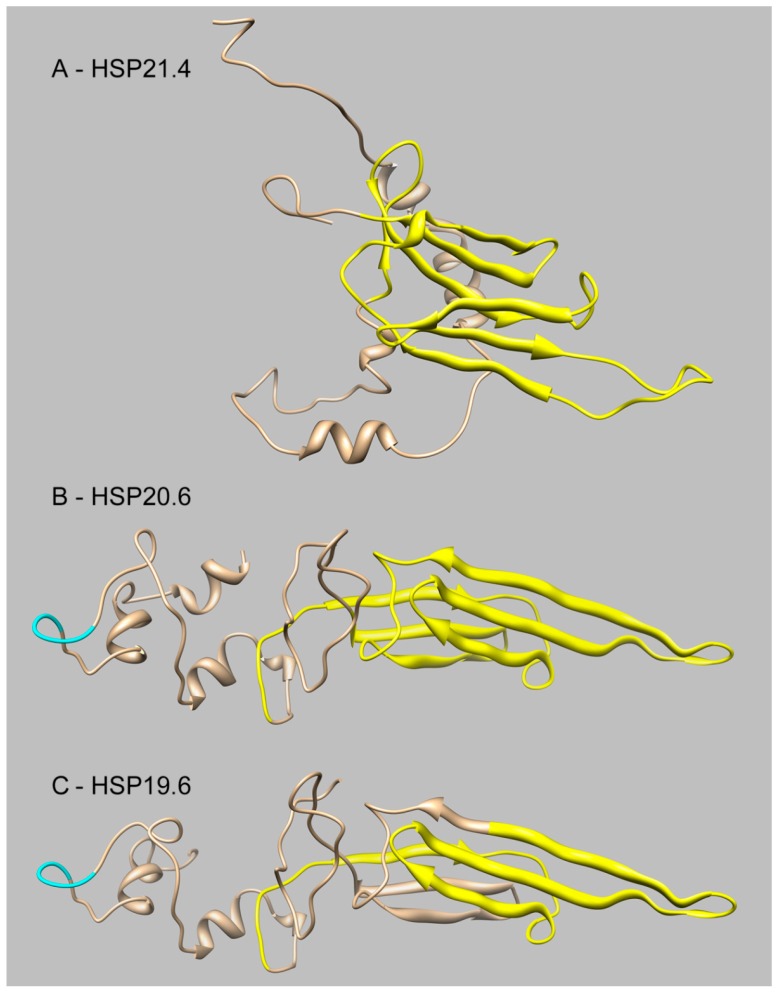
The structural prediction of sHSPs from *Sesamia inferens*. sHSPs include HSP21.4 (**A**); HSP20.6 (**B**); and HSP19.6 (**C**). HSP20 family profiles; Typical α-crystalline domains are indicated in yellow, kinase II phosphorylation sites (TPED) are indicated in cyan, peptides are indicated in brown.

To investigate the structural characteristics of three sHSPs from *S. inferens*, we generated a homology model of *S. inferens* HSP21.4 with Phyre. In this analysis, we used a sHSP from *Taenia saginata* (PDB ID: 2BOL) as a template [30], for which the confidence and coverage are 100% and 88%, respectively (Figure 2A). Homology models of *S. inferens* HSP20.6 and HSP19.6 were deduced with Phyre using *Homo sapiens* αB-crystallin V * (PDB ID: 2YGD) as a template [31] (*S. inferens* HSP20.6: Confidence, 100%, coverage, 91%; HSP19.6: Confidence, 100%, coverage, 93%) (Figure 2B,C).

#### 2.1.3. Phylogenetic Analysis of *S. inferens* sHSPs

**Figure 3:**
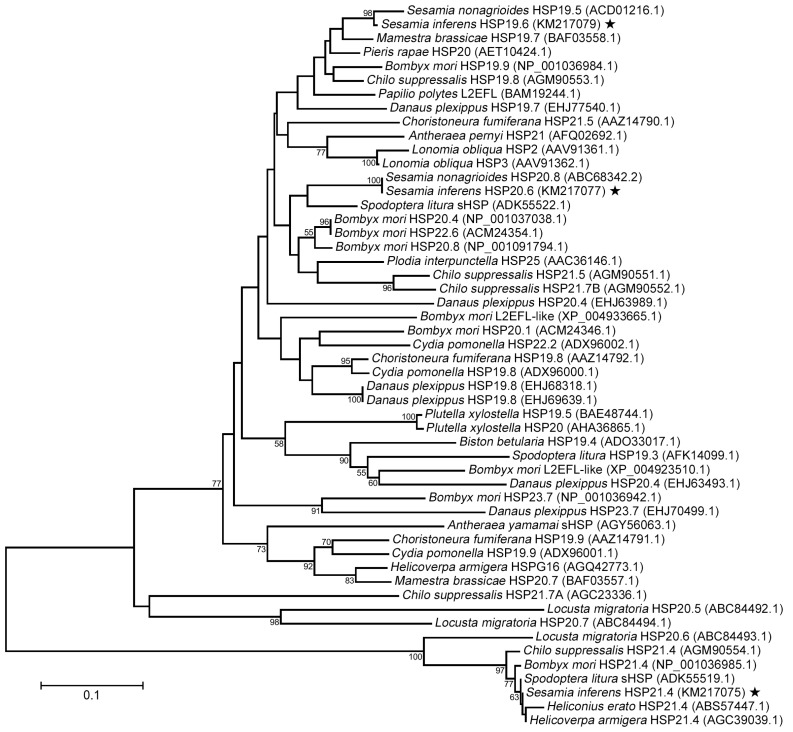
Phylogenetic tree of *Sesamia inferens* and other insect species sHSPs based on neighbor-joining method. The *S. inferens shsps* are labeled with five-pointed star. The *Locusta migratoria* HSP20.5, *L. migratoria* HSP20.6 and *L. migratoria* HSP20.7 were used as the out groups. Origin of sHSP proteins and their GenBank accession numbers are showed in the figure. Numbers on the branches are the bootstrap values obtained from 1000 replicates (only bootstrap values >50 are shown).

The deduced amino acid sequences of the three *shsps* displayed a high degree of relatedness with orthologous proteins reported in other insects. To compare *S. inferens* sHSPs with those from other insects, ClustalX and MEGA 6.06 were used to perform multiple phylogenetic analyses, including neighbor-joining, minimum evolution, maximum likelihood, and maximum parsiomony. The four resulting phylogenetic trees were similar; Thus, only the neighbor-joining tree is shown (Figure 3). The tree could be divided into two major clusters; *S. inferens* HSP19.6 and HSP20.6, which show high sequence similarity, were grouped together in a well-supported cluster (Figure 3). *S. inferens* HSP19.6 showed 97% amino acid identity with *Sesamia nonagrioides* HSP19.5, and *S. inferens* HSP20.6 exhibited 98% identity with *S. nonagrioides* HSP20.8 (Figure 3). *S. inferens* HSP21.4 showed 97%–99% identity with HSP21.4/sHSP from five other lepidopteran species (e.g., *Chilo suppressalis* HSP21.4, *Bombyx mori* HSP21.4, *S. litura* sHSP, *Heliconius erato* HSP21.4, and *Helicoverpa armigera* HSP21.4) (Figure 3).

#### 2.1.4. Genomic Structure of *S. inferens* sHSP Genes

**Figure 4:**
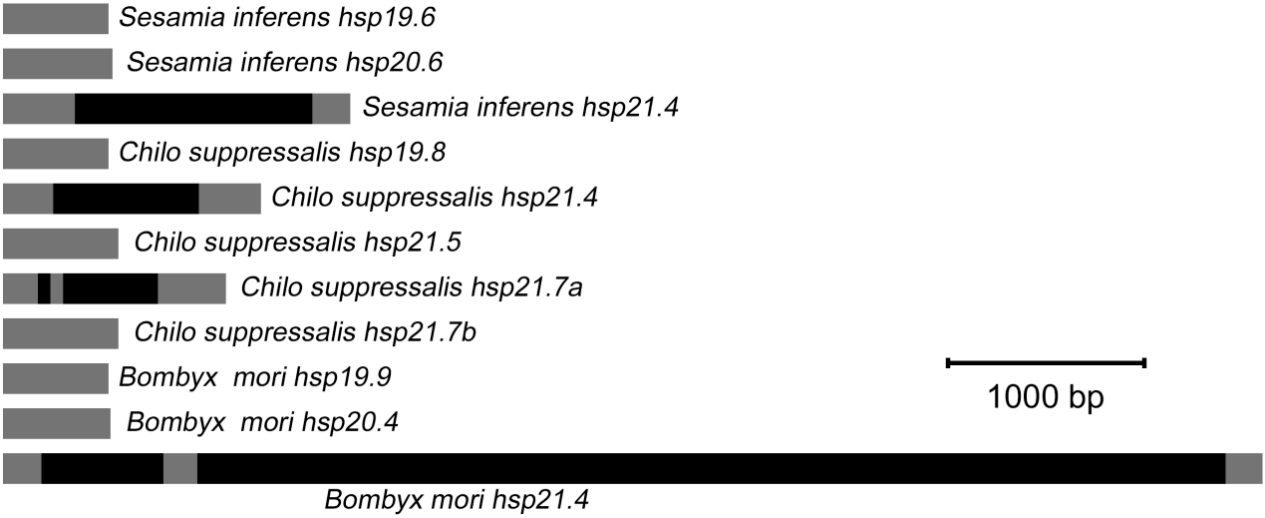
Schematic representation of genomic structures of three lepidopteran insect *shsps*. Origin of *shsps* genomes and their GenBank accession numbers: *Sesamia inferens hsp19.6* (KM217080), *S. inferens hsp20.6* (KM217078), *S. inferens hsp21.4* (KM217076), *Chilo suppressalis hsp19.8* (KC210020), *C. suppressalis hsp21.4* (KC210021), *C. suppressalis hsp21.5* (KC210022), *C. suppressalis hsp21.7A* (KC210023), *C. suppressalis hsp21.7B*, (KC210024), *Bombyx mori hsp19.9* (BGIBMGA004540-TA), *B. mori hsp20.4* (BGIBMGA004541-TA), and *B. mori hsp21.4* (BGIBMGA000944-TA). Gray and black rectangles are used to highlight the exons and introns, respectively.

The genomic DNA sequences of the three *S. inferens* sHSP genes varied in length as follows: 2541 bp for *Sihsp21.4* (GenBank accession no. KM217076), 559 bp for *Sihsp20.6* (accession no. KM217078), and 692 bp for *Sihsp19.6* (accession no. KM217080). The position and size of introns were noted by aligning cDNA with genomic DNA sequences. *Sihsp21.4* had a single 1210 bp intron located within the coding region (472–1681 bp) (Figure 4), and the nucleotide sequences at the intron splice junctions were consistent with the canonical GT-AG rule. Although *S. inferens* HSP21.4, *B. mori* HSP21.4, and *C. suppressalis* HSP21.4 had high amino acid identity (Figure 3), the genomic DNAs differed in the size and/or number of introns (Figure 4). Unlike *Sihsp21.4*, which had a single, 1210 bp intron, *Bmhsp21.4* had two introns (720 and 5531 bp). *Cshsp21.4* had a single intron, but it was smaller than *Sihsp21.4.* The genomic DNAs of *Sihsp20.6* and *Sihsp19.6* lacked introns and were highly homologous to *Cshsp19.8*, *Cshsp21.5*, *Cshsp21.7b*, *Bmhsp19.9*, and *Bmhsp20.4* (Figure 3 and Figure 4).

#### 2.1.5. Expression of Genes Encoding sHSPs in *S. inferens* Tissues

qRT-PCR was used to study the expression profiles of the three *shsps* in *S. inferens*. The presence of single, sharply defined peaks in melting curve analysis of the three *shsps* and six reference genes was confirmed. A standard curve was generated for each gene using eight ten-fold serial dilutions (1×, 10×, 10^2^×, 10^3^×, 10^4^×, 10^5^×, 10^6^× and 10^7^×) of the pooled cDNAs. The PCR efficiency (as calculated from the standard curve) and correlation coefficient (*R*^2^) for each standard curve are shown in Table 2, and the parameters satisfied the basic requirements for quantitative real-time PCR [32].

**Figure 5:**
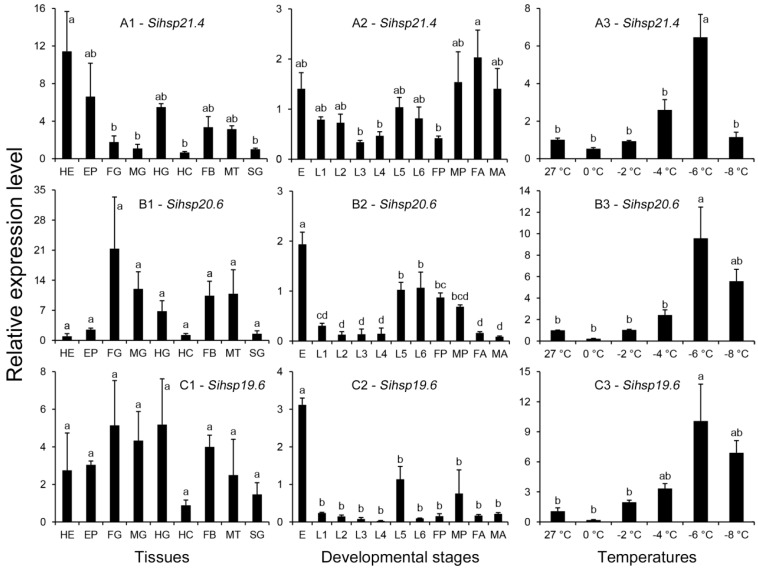
Relative mRNA expression levels of *Sihsp21.4* (**A**); *Sihsp20.6* (**B**); and *Sihsp19.6* (**C**) in different tissues of the 5th instar larvae (**1**), different developmental stages (**2**), and under low-temperature stress (**3**). HE: Head; EP: Epidermis; FG: Foregut; MG: Midgut; HG: Hindgut; HC: Haemocytes; FB: Fat body; MT: Malpighian tubules; SG: Salivary glands; E: Egg; L1: First instar larva; L2: Second instar larva; L3: Third instar larva; L4: Forth instar larva; L5: Fifth instar larva; L6: Six instar larva; FP: Female pupa; MP: Male pupa; FA: Female adult; and MA: Male adult. Each treatment includes three replicates. The data are denoted as mean ± SE. Different letters above bars represent significant differences in the relative expression levels at the 0.05 level (Tukey’s multiple range test).

The three *S. inferens* sHSP genes were expressed in all insect tissues examined, although expression patterns differed amongst tissues. For example, *Sihsp21.4* was expressed more highly in heads than other tissues (*F*_8,18_ = 3.387, *p* = 0.015) (Figure 5A1). However, the expression of *Sihsp20.6* (*F*_8,18_ = 2.006, *p* = 0.105) and *Sihsp19.6* (*F*_8,18_ = 0.935, *p* = 0.512) was not significantly different in the various insect tissues examined (Figure 5B1,C1).

#### 2.1.6. Expression of Genes Encoding sHSPs in Different Developmental Stages

The three *S. inferens* sHSP genes were expressed in all stages of *S. inferens* development, although the expression levels varied widely. Expression of *Sihsp21.4* was highest in female adults (Figure 5B2) and lowest in third instar larvae (*F*_10,22_ = 3.027, *p* = 0.015). Expression of *Sihsp20.6* and *Sihsp19.6* was highest in eggs, and this difference was significant when compared to expression in other developmental stages (*Sihsp20.6*, *F*_10,22_ = 17.301, *p* < 0.001; *Sihsp19.6*, *F*_10,22_ = 16.518, *p* < 0.001) (Figure 5B2,C2).

#### 2.1.7. Expression of Genes Encoding sHSPs in Response to Cold Temperatures

The three *S. inferens* sHSP genes showed similar expression patterns in response to low temperatures. *Sihsp21.4*, *Sihsp20.6* and *Sihsp19.6* were induced in response to cold temperatures. For example, expression levels were significantly higher (6.42–9.56-fold higher) in *S. inferens* exposed to −6 °C for 2 h as compared to insects maintained at 27 °C (*Sihsp21.4*, *F*_5,11_ = 14.706, *p* < 0.001; *Sihsp20.6*, *F*_5,11_ = 7.024, *p* = 0.004; *Sihsp19.6*, *F*_5,11_ = 5.035, *p* = 0.012) (Figure 5A3,B3,C3).

### 2.2. Discussion

Overwintering insects are exposed to temperature stress in nature and must adopt specialized adaptive mechanisms to survive low temperatures. The induction of small heat shock proteins is a potential survival mechanism during temperature stress [19]; However, to our knowledge, the study of sHSPs in *S. inferens* has not been previously undertaken. In the present study, we cloned three members of the sHSP family from *S. inferens*. Analysis of the cDNA sequences and deduced ORFs indicated that *Sihsp21.4*, *Sihsp20.6*, and *Sihsp19.6* encoded proteins containing 187, 183, and 174 amino acids, respectively. The predicted amino acid sequences shared considerable sequence similarity with sHSP from other insects and α-crystallin proteins from vertebrate eye lenses. Phylogenetic analysis indicated that *S. inferens* HSP19.6 and *S. nonagrioides* HSP19.5 clustered with the same group; Whereas *S. inferens* HSP20.6 and *S. nonagrioides* HSP20.8 clustered together in another group (Figure 3). *S. inferens* HSP21.4 and five other lepidopteran HSP21.4 orthologs grouped together in a well-supported cluster, which supports the accuracy of the sequence analysis conducted in the present study. *S. inferens* HSP19.6 and HSP20.6, which show high sequence similarity, grouped together in a well-supported cluster, whereas *S. inferens* HSP21.4 sorted to a different cluster. Thus, *Sihsp21.4* may have evolved differently from *Sihsp20.6* and *Sihsp19.6*. It is interesting to note that sHSP orthologs from *S. litura* and *C. suppressalis* show phylogenetic similarities to the *S. inferens* proteins identified in the present study [19,33].

A negative correlation between intron size and the level of gene expression has been suggested previously; In other words, genes containing smaller introns or lacking introns were more highly expressed than genes containing large or multiple introns [34]. It is also possible that genes either lacking or containing shorter introns may be more sensitive to environmental stresses. Based on chromosomal location and intron number, sHSP genes could be subdivided into two types: Orthologous or species-specific [35]. Thus, the three *shsps* from *S. inferens* could be classified into two groups: Orthologous that contained introns (*Sihsp21.4*) and species-specific forms lacking introns (*Sihsp20.6* and *Sihsp19.6*).

The various functions of small heat shock proteins in insect tissues are not well-understood. One possibility is that sHSPs play important, specialized roles in maintaining normal functioning in different tissues [15]. In this study, *Sihsp21.4* was highly expressed in *S. inferens* heads, which is similar to the high expression of *hsp19.1* and *hsp22.6* reported in *B. mori* heads [35]. The primary nerve center in insects, e.g., the supraoesophageal ganglion and suboesophageal ganglion, is located within the head; Thus, it is possible that sHSPs could protect the nerve centers from external injury. However, *shsps* of *S. litura*, *Apis cerana cerana*, and *C. suppressalis* were expressed at very high levels in malpighian tubules and hindgut tissues [19,33,36]. Malpighian tubules and hindguts function by reabsorbing water, salts, and other substances prior to excretion by the insect; Thus, it remains unclear why *shsps* were highly expressed in these tissues. One hypothesis is that sHSPs protect these tissues from potentially toxic substances [19]. Taken together, our data suggest that different *shsps* play distinct roles in the physiology of *S. inferens*.

sHSPs play important roles in development, including the regulation of insect development [33,37,38]. For instance, *l2efl*, a type of *shsp*, reached a maximum level of expression in the third instar larvae of *D. melanogaster* [39]. However, in *Lucilia cuprina*, expression of *hsp24* was lowest in third instar larvae [37]. Lu *et al.* reported a high level of *Cshsp21.7a* expression in first instar larvae of *C. suppressalis*, whereas the highest expression of *Cshsp19.8*, *Cshsp21.4*, *Cshsp21.5*, and *Cshsp21.7b* was observed in *C. suppressalis* adults; In this study, expression of *Sihsp20.6* and *Sihsp19.6* was highest in insect eggs, whereas *Sihsp21.4* expression was highest in female adults [19]. Thus our data support the hypothesis that sHSPs have evolved specific roles in different stages of insect development.

*Sihsp21.4*, *Sihsp20.6* and *Sihsp19.6* were dramatically up-regulated during low temperature stress, which is similar to *hsp20.4* and *hsp20.8* in *S. litura* and *hsp19.8*, *hsp21.5*, *hsp21.7b* in *C. suppressalis* [19,33]. Previous studies have emphasized the role of sHSPs in modulating thermo-tolerance of insects. However, not all *shsps* are up-regulated during cold stress. For example, the expression of *hsp21.4* and *hsp21.7b* (*C. suppressalis*), *hsp20* and *hsp21.4* (*S. litura*), and *hsp21.4* (*B. mori*) was insensitive to cold stress [19,33,35]. Because the overproduction of HSPs may cause deleterious effects, regulatory controls are critical with respect to maintaining the cost/benefit ratio in organisms expressing these genes [40,41].

More in-depth studies are needed to clarify the role of sHSPs in insect behavior and development. Future investigations will help reveal the underlying physiological mechanisms of *shsps* in *S. inferens*, thus enhancing our ability to implement more effective control measures for this significant pest.

## 3. Experimental Section

### 3.1. Insects

Populations of *S. inferens* were collected from a suburb of Yangzhou (32°39'N, 119°42'E), located in the Jiangsu province. The pink stem borers were reared for more than 3 generations in environmental chambers maintained at 27 ± 1 °C with a 16:8 (light/dark) photoperiod and 60%–70% relative humidity as described previously [42].

### 3.2. Reverse Transcription Polymerase Chain Reaction (PCR) and Rapid-Amplification of cDNA Ends (RACE)

Total RNA was extracted from *S. inferens* using the SV Total RNA isolation system (Promega, Madison, WI, USA) and then treated with DNase I. The integrity of RNA was verified by comparing RNA bands in gels stained with ethidium bromide. RNA purity was analyzed at 260 and 280 nm using a spectrophotometer (Eppendorf BioPhotometer plus, Eppendorf, Germany). cDNA copies of genes encoding sHSPs were synthesized using oligo(dT)_18_ primers (Fermentas, Helsingborg, Sweden). Degenerate primers for PCR were designed using consensus sequences of *shsps* obtained from several lepidopteran insects; These sequences were previously deposited in GenBank (Table 1). Degenerate primers of *Sihsp21.4* were designed using consensus sequences of *H. armigera hsp21.4*, *Spodoptera litura hsp21.4* and *C. suppressalis hsp21.4*; Degenerate primers of *Sihsp20.6* were designed using consensus sequences of *S. nonagrioides hsp20.8*, *S. litura hsp20.4* and *B. mori hsp20.4*; Degenerate primers of *Sihsp19.6* were designed using consensus sequences of *S. nonagrioides hsp19.5*, *B. mori hsp19.5* and *Plutella xylostella hsp19.5*. And the amino acid regions used to design degenerate primers are conserved regions of each gene. Full-length cDNAs were obtained using 5' and 3' RACE (SMARTer™ RACE, Clontech, Palo Alto, CA, USA). Primers for RACE were designed based on partial sequence information derived from *shsps* cDNA fragments (Table 1). Complete sequences of intact ORFs were confirmed by 5' RACE cDNA. Products were purified using the AxyPrep™ DNA Gel Extraction Kit (Axygen, Union City, CA, USA), cloned into pGEM-T Easy Vector (Promega), and then transformed into *Escherichia coli* DH5α cells for subsequent sequence analysis.

**Table 1:** Primers used for gene clone and verification.

Primer Name	Primer Sequences (5'–3')	Amplicon Size (bp)	Purpose
hsp21.4DP-F	ATGGARGAAGAAATGASAARTT	241	Intermediate fragment amplification
hsp21.4DP-R	TCGACHGTCTTVACRACGAT		
hsp20.6DP-F	CCTMGCCGYCTGDTGGAYCARC	467	
hsp20.6DP-R	TCCTTGATCTCCTTGCGVACGG		
hsp19.6DP-F	AAGTBAACCTDGACGTGCAGCATT	296	
hsp19.6DP-R	TTTCACCTCCTTGCGCACTGGT		
hsp21.4RACE-5'	CTTCAATGACTTGCCGTCGCCCTC	418	Rapid-amplification of cDNA ends (RACE)
hsp21.4RACE-3'	AGCACAGTGACAGCAGACAGTTGGC	1062	
hsp20.6RACE-5'	ATTCCACGGACTCGGGTTCCACGC	510	
hsp20.6RACE-3'	TTGCTGCTGGACCTTTGCTGACGA	610	
hsp19.6RACE-5'	TCTGGTAAAGCGTAGCGGCGGGT	502	
hsp19.6RACE-3'	GGCAGTTTACCCGCCGCTACGCT	327	
hsp21.4cDNA-F	ATGGGGAGTACTGCCTTG	963	Verification of open reading frame (ORF)
hsp21.4cDNA-R	TGCCTGAATACATCCCTTA		
hsp20.6cDNA-F	AAGATGTCTCTGTTGCCA	559	
hsp20.6cDNA-R	CACTTTACTTCTTTTCCTTT		
hsp19.6cDNA-F	GTGCGAAACAAGTACAAAGC	692	
hsp19.6cDNA-R	CAAAGAGAACACTGAAAGGAAT		
hsp21.4DNA1-F	TGTCTGCTGTAGAGTGCGTAG	372	Verification of genome
hsp21.4DNA1-R	TTGAACTCGCCCCTGATC		
hsp21.4DNA2-F	TGGGACAGCTTGAACTCG	1529	
hsp21.4DNA2-R	AGTGCTTCTGGATGGGGA		
hsp21.4DNA3-F	CGACAGGAACATCCCCATCCAGAA	697	
hsp21.4DNA3-R	TATTGACATCTAAAACATTCGTAAC		
hsp20.6DNA-F	AAGATGTCTCTGTTGCCA	559	
hsp20.6DNA-R	CACTTTACTTCTTTTCCTTT		
hsp19.6DNA-F	GTGCGAAACAAGTACAAAGC	692	
hsp19.6DNA-R	CAAAGAGAACACTGAAAGGAAT		

### 3.3. Characterization of Genomic DNA

The genomic DNA of *S. inferens* was extracted using the AxyPrep™ Multisource Genomic DNA Kit (Axygen Biosciences, Union City, CA, USA). Specific primer pairs (Table 1) were designed to amplify genomic fragments based on analysis of full-length cDNAs. The products were purified using the AxyPrep™ DNA Gel Extraction Kit (Axygen), cloned into pGEM-T EasyVector (Promega), and transformed into *E. coli* DH5α for sequence analysis.

### 3.4. Tissues Samples

The larvae selected for analysis were similar in size and randomly assigned to experimental groups. Each group contained ten larvae, and each experiment was repeated three times. Larvae were anesthetized on ice prior to dissection. The head, epidermis, fat body, foregut, midgut, hindgut, malpighian tubules, haemocytes, and salivary glands were collected from larvae and rinsed with a 0.9% sodium chloride solution. The samples were frozen immediately in liquid nitrogen and stored at −70 °C prior to real-time PCR analyses.

### 3.5. Samples Representing Developmental Stages and Sex

Samples included egg masses, the first, second, third, fourth, fifth and sixth instar larvae, male and female pupae, and one-day-old male and female adults; Samples were randomly selected for the experiment. The samples were frozen immediately in liquid nitrogen and stored at −70 °C until needed for analyses.

### 3.6. Cold Tolerance Samples

In this experiment, larvae representing the fifth instar were placed individually in glass tubes, and groups of ten were then exposed to various temperatures (−8, −6, −4, −2 and 0 °C) for 2 h in a constant-temperature incubator (DC-3010, Jiangnan Equipment, Changzhou, China). The larvae were recovered at 27 ± 1 °C for 2 h, after which surviving larvae were frozen in liquid nitrogen and stored at −70 °C. A set of larvae maintained at 27 ± 1 °C was regarded as a control group. Each treatment included at least three surviving larvae.

### 3.7. Quantitative Real-Time PCR

Total RNA was extracted using the methods described above for reverse transcription PCR and RACE. RNA (0.5 μg) was reverse-transcribed into first-strand cDNA using the Bio-Rad iScript™ cDNA Synthesis Kit (Bio-Rad, Hercules, CA, USA). Real-time PCR reactions were performed in a 20 μL reaction volume comprised of 10 μL Bio-Rad iTaq™ Universal SYBR^®^ Green supermix (Bio-Rad, 2×), 1 μL of each gene-specific primer (10 μM) (Table 2), 2 μL of cDNA template, and 6 μL of ddH_2_O. Reactions were carried out using a CFX-96 real-time PCR system (Bio-Rad) under the following conditions: 3 min at 95 °C, 40 cycles of denaturation at 95 °C for 30 s, and annealing at the Tm for each gene (30 s; Table 2). Each treatment included three replicates, and each reaction was run in triplicate.

### 3.8. Data Analysis

ORFs were identified using ORF Finder (http://www.ncbi.nlm.nih.gov/gorf/gorf.html). The deduced amino acid sequences were aligned using CLUSTAL X1.83 [43]. Sequence analysis tools of the ExPASy Molecular Biology Server (Swiss Institute of Bioinformatics, Basel, Switzerland) were used to analyze the deduced sHSP sequences, including Translate, Compute pI/*M*_W_, and Blast. Amino acid sequences were used to estimate phylogeny using neighbor-joining, minimum evolution, maximum likelihood, and maximum parsimony methods. Phylogenetic trees were constructed with 1000 bootstrap replicates using MEGA version 6.06 (Tempe, AZ, USA) [44].

Homology models were generated using Protein Homology/analogy Recognition Engine software version 2.0 (http://www.sbg.bio.ic.ac.uk/~phyre2/html) [45]. The Chimera tool was used to visualize the 3D coordinates for the atoms in the predicted protein models [46].

**Table 2:** Primers used for qRT-PCR.

Gene	Primer Sequences (5'–3')	Amplicon Size (bp)	PCR Efficiency	*T*_m_ (°C)	*R*^2^
hsp21.4qRT-F	TGGCTGACAGTGGTCTGAAAA	196	91.6%	60.1	0.996
hsp21.4qRT-R	GTGGTGCTGCTAGTTGTGCTT				
hsp20.6qRT-F	GCATCAAGACTGACGGAGATAAG	111	99.5%	60.1	0.994
hsp20.6qRT-R	GTTTGCCTTCCACCACAATG				
hsp19.6qRT-F	CGAAGGTAAACACGAGGAGAAG	132	102.7%	58.2	0.970
hsp19.6qRT-R	GTCAAAACGCCGTCAGAAGA				
RPS13qRT-F	TGGTAAGGGTATCTCCCAATCA	75	93.5%	60.1	0.994
RPS13qRT-R	TCGTCAGCAGTCAGTTTCAGC				
RPS20qRT-F	CTCATCAATGGAGCCAAGAAAC	162	102.0%	60.1	0.986
RPS20qRT-R	GTGCAGGTCAATGACACGCT				
EF1qRT-F	GTCGCTTTCGTACCCATTTCT	86	97.4%	56.6	0.994
EF1qRT-R	ACAGTCCATCCCTTGAACCAG				
18SqRT-F	CAACACGGGAAATCTCACCA	115	107.3%	55.6	0.996
18SqRT-R	GACAAATCGCTCCACCAACTAA				
GAPDHqRT-F	GGTCATCTCCAACGCTTCCT	166	95.0%	56.6	0.993
GAPDHqRT-R	ACGTCCATCACGCCACAAT				
TUBqRT-F	TTGCTACAGAACCCTCAAAGTGC	159	104.4%	59.2	0.985
TUBqRT-R	AGACGTGGGAACGGAACCAT				

qRT-PCR data were analyzed using the Bio-Rad CFX Manager™ 3.1 software (Bio-Rad). The threshold cycle (*C*_t_ value) denotes the cycle at which the fluorescent signal first shows significant difference with respect to the background. All biological replicates were used to calculate the average *C*_t_ values. Relative expressions were calculated using the 2^−ΔΔ*C*t^ method [47]. Three genes (*RPS13*, *RPS20* and *EF1*) were used for normalizing gene expression in different tissues. *18S rRNA*, *EF1* and *GAPDH* were used as reference genes in different developmental stages and sexes. *18S rRNA*, *RPS20* and *TUB* were used for normalizing gene expression at different temperatures. The means of the reference genes were used as normalization under different experimental conditions [48]. The above-mentioned reference genes were previously validated in a research study that has been submitted elsewhere. Tukey’s test was conducted for statistical analysis using PASW Statistics 18.0 (SPSS Inc., Chicago, IL, USA).

## 4. Conclusions

In conclusion, we cloned three genes encoding sHSPs from *S. inferens*. The structure of these genes was examined, and we analyzed their expression in different tissues and stages of insect development. Our results also indicate that expression of the three *shsps* is modulated in response to cold stress.
